# Athletes’ medical preventive behaviors: the case of oral health and ultraendurance trail runners

**DOI:** 10.1186/s12903-024-04492-3

**Published:** 2024-07-11

**Authors:** Amélie Doré, Romain Jacq, Anne-Charlotte Bas

**Affiliations:** 1 U1018, Inserm, Centre de recherche en Épidémiologie et Santé des Populations, University of Paris Saclay, UVSQ, Villejuif, France; 2 Dental Department, UFR Santé, University of Rouen Normandy, 22 boulevard Gambetta, Rouen Cedex, 76183 France

## Abstract

Ultra-endurance runners are particularly exposed to caries risk factors due to the continuous intake of sugars in liquid or sticky forms during long-distance exercise. The risk of caries increases due to a failure to perform oral hygiene during races. Ultra-endurance runners had good oral health status despite these particularities. Our hypothesis is that high compliance with preventive oral health recommendations (toothbrushing and preventive or regular dental visits) counterbalance the risks associated with their exposure to caries. We aimed to gain a better understanding of preventive dental behaviors in ultra-endurance runners. We then studied the determinants of two major recommendations: (1) visiting a dentist for preventive check-ups and (2) visiting a dentist during the year. 37% of the total sample reported patterns of both preventive dental visits and recent visits. Early visits (ORa = 4.8***), good oral health (ORa = 8.8**) and tooth brushing (ORa = 2.2**) were associated with preventive dental visits, but there was no significant influence of snacking or weekly work hours. The ultra-endurance race frequency was associated with early dental visits despite equal needs. Most risk-control behaviors were associated with each other, indicating that they are all-or-nothing behaviors. Individual prevention strategies implemented at the dental office may not be as effective as they predominantly target individuals who are already aware of and interested in preventive care. Instead, developing targeted primary prevention strategies that are accessible at race venues, such as stands, villages, or food supply points, could be more effective.

**Supplementary Information:**

The online version contains supplementary material available at 10.1186/s12903-024-04492-3.

## Key points

• Ultra-endurance runners are particularly exposed to caries risk factors, but they still have good dental health status.

• More runners are involved in the races more they practice early dental visits.

• Preventive dental behaviors co-exist in ultra-endurance runners’ population and potentiate each other.

**Supplementary Information:**

The online version contains supplementary material available at 10.1186/s12903-024-04492-3.

## Context

Mountain and trail racing has recently gained popularity [1, 2]. The International Trail Running Association registered 1,600,000 runners and 23,880 races in 2019. These races occur on natural terrain in various kinds of environments, such as mountains and forests, and mostly on off-road surfaces. These running races last more than six hours, and athletes have limited provisions that are selected according to their security and utility in enhancing (energy) or restraining (weight) performance. Runners train regularly and intensively and run very long races [3]. This entails specific nutritional needs involving frequent, regular intake of sugary foods during exercise, whether during training or races [4–7]. This intake during the autonomy period increases athletes’ exposure to oral health risk factors, such as caries development [8]. The diet also has a significant impact on tissue inflammation, particularly oral tissues, which can affect overall health as well [9, 10]. The first explorative study showed that nutritional intake during exercise involved foods likely to induce tooth decay, such as energy bars, sugar-rich gels, and fruit confectionaries, with frequent snacking. The presence of sugar on teeth favors the development of bacteria that cause tooth decay [11]. Nonetheless, the dental health of 84% of the participants was “good” or “very good” [8]. This is quite bigger than in general population, knowing only 57.5% of the adult French population considers themselves to have “good” or “very good” oral and dental health [12]. Thus, this study uncovered surprising findings that may intrigue dentists: despite facing significant risks of dental caries, these athletes maintain satisfactory overall oral health. One possible explanation for this paradox lies in a dedicated health behavior ingrained in their lifestyle, underscoring the importance of consistent dental care practices. This proactive approach includes adherence to dental check-ups and regular tooth brushing. The participants, heavily engaged in intensive training as part of their pursuit of peak performance, prioritize a healthy lifestyle that encompasses meticulous oral hygiene. Their commitment to health preferences likely drives their compliance with dental care routines. Building upon these observations, our study aims to examine the interplay of dental health habits, hypothesizing a robust control of caries protective factors among ultra-endurance runners. We first justify this hypothesis in the following paragraphs, relying on existing literature.

As in global health, preventive oral health behaviors are characterized by interactions among individuals and social and environmental determinants [13]. The influence of these determinants on health status follows the additive logic of the exposome, i.e., the integration of all the exposures—of whatever kind—to which an individual is subjected during their lifetime [14]. These models imply that established health habits play a particularly important role on health status [15]. These habits or preventive health behaviors reveal health preference, health education and risk aversion [16]. Risk aversion refers to an economic agent’s tendency to prefer certainty to uncertainty. People with a strong aversion to risk tend to avoid it, while people with relatively high risk aversion are more likely to adopt healthy behaviors as use of health preventive behaviors [17]. Some psychologists and economists believe that perceptions of risk and chances of cure, risk preference and time orientation may also explain disparities in screening [18, 19]. The social gradient is expressed in health preferences, poor or unqualified people tend to undergo screening less than their wealthier or better-educated counterparts [18, 19].

In a recent systematic review, the occurrence of mental health issues among ultrarunners was found to range between 11.5% and 18.2% concerning the risk of exercise addiction [20] This addictive behavior seems to lead to overtraining among ultrarunners. Many of these individuals seek to train more intensely and for longer periods, with the aim of improving their performance and physical capacity. Nutritional intake and oral health are two important elements of this quest. On one hand, to support long and intense efforts [4–7], and on the other hand, to maintain a strong, healthy body capable of protecting itself from injuries [9, 10, 21]. Ultrarunners may adopt various preventive behaviors as part of their efforts to enhance their sporting and physical performance. While in total control of their bodies and their performance, these individuals practice risk aversion by putting an oral health routine in place with the aim of achieving performance rather than a state of health [20, 22]. In the context of sports injury prevention, Verhagen et al. documented the relationship between preventive health behavior and sports behavior. They showed that taking health risks is not only accepted to improve performance but also accepted without realizing it, as the risk taken is a force of habit [23]. The risk could project onto all health outcome terms [24]. Athletes have good knowledge of eating habits, nutrition, body composition, and physical performance [25]. For example, marathon runners organize their nutritional intake according to the performance gains they hope to achieve [21]. The risks relating to the characteristics of this directed diet do not seem to be accounted for, are considered only to a limited extent, or are considered acceptable.

There is a growing body of literature on athletes’ health behaviors, but very few studies focus on dental health, despite the recent identification of significant risk factors. The study’s objective is to identify the determinants of preventative dental behavior in a population of ultraendurance trail runners. We focused on two preventive dental behaviors: (1) visiting a dentist for preventive check-ups and (2) visiting a dentist during the year.

## Materials and methods

This was a descriptive epidemiological study based on a cross-sectional self-report survey. The survey was conducted as part of the mission of the INSERM laboratory U1042 to study the health of runners participating in the *Ultra-Trail des 4 Massifs* (UT4M) race. The UT4M is a series of 14 ultratrail races ranging from 40 to 169 km in length that are organized every summer in the Alps.

### Questionnaire and sample

The target population included 3 000 ultra-endurance runners registered for the UT4M races. The questionnaire contained several sections, each dedicated to various aspects of runners’ health concerns. The oral health section of the questionnaire was developed and included in the overall project by the authors (RJ and ACB), with support from current literature on declarative oral health statements [8]. The questionnaire is available as supplementary material. It was distributed to candidates via email from March to June 2020. The survey was anonymous and voluntary. The informed consent was obtained as a condition to access the questionnaire from all subjects and/or their legal guardian(s). The data were collected and stored on Google Forms.

We selected the 263 patients who completed the oral health section of the questionnaire (1/12th of the target population, after removing individuals with identifiable data collection errors, duplicates, and aberrant values from the sample). The data were collected in light of the concomitant cancellation of the 2020 UT4M races due to the impossibility of enforcing health precautions linked to coronavirus disease 2019 (COVID-19). We could not ensure the representativeness of the sample because of selection bias. We discuss these biases in the primary study, noting that the samples before and after selection had similar proportions of women, a similar age structure and similar durations of training per week.

The study was conducted in accordance with the Helsinki Declaration and approved by the INSERM’s ethics committee and the National Agency for the Safety of Medicines and Health Products (ANSM) under the ID-RCB number: 2018-A01092-53,

### Variables

The variables retained for this study related to personal and sporting characteristics, health status, and preference for oral disease prevention. We selected variables that show significant impact The personal and sporting variables were as follows: age, sex, yearly frequency of races (< 4 races, 4 to 8 races, > 8 races), and weekly working hours (< 35 h, 35–45 h, > 45 h).

The health variable retained for the participants was dental health status. This variable concerned self-reported health status, with the respondent indicating whether their status was “very good/fair/poor”.

The variable concerning risk factors for tooth decay associated with nutrition was a variable that combined nutritional risk factors for tooth decay among athletes: responses of “in excess/not in excess” were recorded.

The variables relating to oral health routines were the frequency of daily tooth brushing (< 2 times, 2 times, > 2 times), the duration since the last dental visit (more or less than one year), and the purpose of the visit (prevention or treatment).

### Data analysis

After a descriptive analysis of the data, a bivariate analysis was performed to evaluate the relationships across all variables and theses related to dental visits. The analyses enabled us to test the independence between prevention factor variables and the purpose of the last dental visit and between prevention factor variables and the duration since the last dental visit. The results are presented in Table 1. These tests guided us in the construction of 2 multivariate logistic regressions for (1) the purpose of the last dental visit and (2) the duration since the last dental visit. The explicative variables were selected on the basis of statistically significant associations and the relevance of certain adjustment variables. The results are presented in Table 2 in the form of adjusted odds ratios (ORs), p values and 95% confidence intervals (95% CIs).

**Table 1:** Descriptive statistics: total, two-way tables according to the variables related to the last dental visit (*N* = 263)

	Total	Purpose of the last dental visit		Duration from the last dental visit	
		Treatment	Prevention	≥ 1 year	< 1 year
**Purpose of the last dental visit**				χ² =35.198***	
Treatment	100 (38.02%)	-	-	78 (78.00%)	22 (22.00%)
Prevention	163 (61.98%)	-	-	66 (40.49%)	97 (59.51%)
**Duration from the last dental visit**		χ² =35.198***			
≥ 1 year	144 (54.75%)	78 (54,17%)	66 (45.83%)	-	-
< 1 year	119 (45.25%)	22 (18.49%)	97 (81.51%)		
**Sex**		χ² = 6.664*		χ² =1.541	
Men	210 (79.85%)	88 (41.90%)	122 (58.10%)	119 (56.77%)	91 (43.33%)
Women	53 (20.15%)	12 (22.64%)	41 (77.36%)	25 (47.17%)	28 (52.83%)
**Age**		pwcorr. =0.056		pwcorr.=0.173**	
Years (mean)	41.882 (0.667)	41.11 (1.172)	42.35 (0.801)	40.19 (0.885)	43.93 (0.984)
**Dental Status**		χ² =14.193**		χ² =6.963*	
Poor	15 (5.70%)	12 (80.00%)	3 (20.00%)	13 (86.67%)	2 (13.33%)
Fair	162 (61.60%)	63 (38.89%)	99 (61.11%)	88 (54.32%)	74 (45.68%)
Very good	86 (32.70%)	25 (29.07%)	61 (70.93%)	43 (50.00%)	43 (50.00%)
**Weekly working hours**		χ² =3.360		χ² =4.248	
< 35 h	58 (22.05%)	21 (36.21%)	37 (63.79%)	25 (43.10%)	33 (56.90.%)
35–45 h	147 (55.89%)	51 (34.69%)	96 (65.31%)	84 (57.14%)	63 (42.86%)
> 45 h	58 (22.05%)	28	30 (51.72%)	35 (60.34%)	23 (39.66%)
**Yearly frequencies of race(s)**		χ² =0.417		χ² = 4.797*	
0–3	67 (25.19%)	24 (34.85%)	43 (65.15%)	37 (54.55%)	30 (45.45%)
4–8	138 (52.67%)	54 (39.13%)	84 (60.87%)	83 (60.14%)	55 (39.86%)
> 8	58 (22.14%)	23 (39.66%)	35 (60.34%)	25 (43.10%)	33 (56.90%)
**Daily toothbrushing**		χ² =5.918*		χ² =0.376	
Less than 2	50 (19.01%)	26 (52.00%)	24 (48.00%)	29 (58.00%)	21 (42.00%)
Twice	174 (66.16%)	58 (33.33%)	116 (66.67%)	93 (53.45%)	81 (46.55%)
More than 2	39 (14.83%)	16 (41.03%)	23 (58.97%)	32 (56.41%)	17 (43.59%)
**Excessive sugar exposure**		χ² =0.463		χ² =0.021	
No	114 (43.35%)	46 (40.35%)	68 (59.65%)	63 (55.26%)	51 (44.74%)
Yes	149 (56.65%)	54 (36.24%)	95 (63.76%)	81 (54.36%)	68 (45.64%)
**Total**	100%	100 (38.02%)	163 (61.98%)	144 (54.75%)	119 (45.25%)

Key : * *P*-value ≤ 0.1 ; ** *P*-value ≤ 0.05 ; *** *P*-value ≤ 0.01

**Table 2:** Multivariate analysis, for (1) the purpose of the last dental visit (Treatment (ref.) vs. Prevention) and (2) the duration since the last dental visit ( = > 1year (ref.) vs. < 1 year)

	(1) Purpose of the last dental visit		(2) Time from the last dental visit	
	ORa (*p*-value)	CI 95%	ORa (*p*-value)	CI 95%
**Purpose of last dental visit** *– Ref = Treatment*				
*Prevention*	-	-	4.981 (0.000)	[2.662; 9.317]
**Time from the last dental visit** *– Ref = ≥ 1 year*				
*< 1 year*	4.838 (0.000)	[2.609; 8.973]	-	-
**Sex** - *Ref = Men*				
*Women*	2.654 (0.019)	[1.176; 5.990]	1.054 (0.881)	[0.526; 2.114]
**Age** *(in years)*	1.014 (0.348)	[0.985; 1.427]	1.041 (0.004)	[1.013; 1.074]
**Dental Status** – *Ref = Poor*				
*Fair*	5.305 (0.023)	[1.262; 22.300]	3.583 (0.140)	[0.657; 19.544]
*Very good*	8.766 (0.004)	[1.962; 39.156]	4.441 (0.094)	[0.775; 25.446]
**Weekly working hours -** *Ref = < 35 h*				
< 35 h	1.682 (0.176)	[0.792; 3.568]	0.653 (0.229)	[0.326; 1.307]
35–45 h	0.929 (0.869)	[0.388; 2.224]	0.652 (0.322)	[0.279; 1.521
**Yearly frequencies of race(s)** – *Ref = < 4*				
4–8	0.830 (0.603)	[0.412; 1.672]	0.888 (0.726)	[0.458; 1.722]
> 8	0.654 (0.317)	[0.271; 1.525]	2.083 (0.076)	[0.925; 4.687]
**Daily toothbrushing** *- Ref = < twice a day*				
*twice a day*	2.174 (0.036)	[1.051; 4.500]	0.944 (0.879)	[0.452; 1.971]
*> twice a day*	1.216 (0.691)	[0.464; 3.186]	0.903 (0.834)	[0.347; 2.350]
**Excessive sugar exposure** *- Ref = No*				
*Yes*	1.381 (0.277)	[0.771; 2.471]	1.199 (0.525)	[0.684; 2.100]
*Constant*	*0.033*		*0.017*	
*Pseudo R2*	*0.180*		*0.156*	

These analyses were conducted with STATA/SE 16® software.

## Results

### Descriptive statistics

A total of 80% of the participants were male, and the mean age was 42 years (SD 10.8). The age range was 21 to 70 years. A total of 56% of the participants worked between 35 and 45 h per week (Table 1).

The reported oral and dental health status was also very satisfactory, with 61.6% of the participants reporting fair health and 32.7% reporting “very good” health. Only 5.7% of the runners reported having poor oral health. A total of 52.7% of the participants registered for 4 to 8 races per year. 15% of the participants said they did not brush enough.

For 62% of the participants, the purpose of their last dental appointment was to prevent oral disease. Among the participants, 60% had visited the dentist within the year. This means that 37% of the total sample reported both a pattern of preventive dental visits *and, at the same time*, sufficiently frequent visits.

### Multivariate analysis: purpose of the last dental visit

There was a positive correlation between the runners’ oral health status and dental visits for prevention purposes (Table 2). Runners who reported good dental health were approximately 8.7 times more likely to report preventive dental visits than were those who reported poor dental health (ORa = 8.766, CI 95% = [1.962; 39.156]).

Women were 2.6 times more likely to report a preventive visit than men were (ORa = 2.654, CI 95% = [1.176; 5.990]). This last result is in line with the literature on health preferences (Lipsky et al., 2021).

Patients who visited a dentist within one year were 4.8 times more likely to visit for preventive reasons (Ora = 4.838, CI 95% = [2.609; 8.973]). Sufficient tooth brushing was associated with a significantly greater probability of having a preventive dental visit (Ora = 2.174, 95% CI= [1.051; 4.500]). Exposure to sugar did not appear to have a significant impact on dental visit purpose’s. These results showed that the preventive behavior of visiting a dental surgeon is associated with the best-known oral hygiene behavior, namely, regular tooth brushing.

### Multivariate analysis: duration since the last dental visit

The determinants of the duration since the last dental visit differed from the purpose of the visit (Table 2). Recent visits were associated with preventive visits, very good oral health (ORa = 4.441; 95% CI= [0.775; 25.446]) and sustained participation in trail races (ORa = 2.083; 95% CI= [0.925; 4.687]). The absence of a significant association with the intermediate modalities of the health status variables can be attributed to an antagonistic effect resulting from having a recent visit because of the need for urgent dental care. The duration since the last visit variable is thus a less effective proxy for oral health prevention behavior than the nature of the dental visit.

## Discussion

Our results showed that ultraendurance trail runners are particularly adherent to dental prevention visits. 37% of the total sample reported both a pattern of dental checkups and recent dental visits. Early visits, good oral health and tooth brushing were associated with dental checkups. The frequency of ultraendurance trail races was associated with early visits for equal needs. However, there was no significant association with regular sugar intake, which is specific to this population.

We first discuss the position of the results, comparing the data from the general population. Our sample exhibited better oral health and dental habits than did the general population, with more people considering themselves to have “good” or “very good” oral and dental health [12] but also more people brushing their teeth as it’s recommended (81% versus 71% in the French population) [26]. Weekly working hours had no significant influence on dental visits. Our sample does have a different structure than the general population since the majority were men aged 30 to 50 years. However, this representation bias in the sample has a tendency to underestimate risk factors since men, who were overrepresented in this study, generally have poorer dental health, brush their teeth less frequently and have fewer preventive visits with their dentists [27, 28].

Good dental status is associated with both preventive and recent dental visits. This finding is fortunately in line with the World Health Organization (WHO) international guidelines on oral health [29]. Indeed, regular checkups allow dentists to see if there are any dental problems and help individuals maintain their oral health. The efficiency of preventive observance seems to increase since dental checkup attendance and tooth brushing are also associated. This is observation is of importance because it strengthens the literature on oral health prevention and highlights the effectiveness of a health behavior in counteracting a caries risk that is known to increase through several factors, namely the continuous intake of sugary and sticky foods and the absence of brushing for more than an hour after these intakes. The accumulation of beneficial behaviors in some individuals is also to be contrasted with the lack of prevention in others, underscoring the challenged state of oral health practices. To improve the dental health of ultra-endurance runners, it is therefore necessary to first target those who do not follow any of the current recommendations.

As if there is a threshold, only the most invested in races have an increased probability of a recent dental visit. The search for improved performance can lead runners to pursue better health and hypercontrol of associated factors [20]. This relationship between preventive and sports behaviors can thus be associated not only with individual and environmental factors such as perfectionism or social pressure related to performance but also with body and aesthetic stereotypes [30]. Performance and aesthetics lead to very strict control of nutritional intake by ultra-endurance athletes. There are preconceived notions that ultraendurance trail runners must have a thin and light body to perform [31]. These nutritional standards followed a perfectionist and well-informed population imply knowledge of the caries risk associated with the associated sweet and sticky food intake. Since these inputs are necessary to support performance, this risk is perceived as acceptable and can trigger compensatory behavior through good adherence to the principles of prevention in oral health, such as regular dental checkups [20]. Competitions are an opportunity to share dental health and nutritional information, such as body norms or eating attitudes, with peers [32–34].

Finally, some limitations and strength of this study should be highlighted. First, there is inherent selection bias in the sample studied, which may restrict the generalization of the results to a wider sportspeople population. Our sample presented characteristics that are similar to those of a study in 2023 on French ultraendurance trail runners [35]; thus, we can be optimistic regarding its representativeness for the discipline. Moreover, the work carried out in this study focused mainly on prevention and oral health behaviors in relation to sports habits, but other factors, such as health insurance, could also influence the oral health of ultraendurance trail runners [36]. Therefore, a broader survey, including a more diverse population and other relevant variables, is needed to complete this study.

The strengths of this study lie in its integration of oral health inquiries into a survey of ultra-endurance runners’ general health. We capitalized on a substantial study population, notably given the scarcity of dental health data, especially regarding preventive health behaviors. These areas remain poorly understood and studied, despite their increasing relevance, driven by the desire to move beyond post-destructive dentistry and the rising number of long-distance runners. Our research elucidates compensatory behaviors for risk-taking and their efficacy, while also scrutinizing the body control behaviors of elite athletes. We delved into these aspects relying on the literature. Similarly, as mentioned in the previous paragraph, this highlights the necessity for a questionnaire to comprehensively assess athletes’ perceptions of their bodies, sports practices, and aspirations – including inquiries about beliefs related to oral health and dental care, which could prove beneficial.

## Conclusion

Preventative oral health behaviors involving visiting a dentist and regular brushing habits can be observed among ultra-endurance runners and are associated with good dental health. This highlights the importance of ultraendurance trail runners’ engagement in preventive practices to maintain their bodily functions, including their oral health. The specificities of this behavior can be explained by runners’ performance goals and their aversion to the risk of disease. The lack of impact of nutrition on these prevention habits shows the ability of individuals to develop indirect compensatory behaviors in the face of the proven caries risk that they do not want to control by changing their nutritional habits.

The targeting of the most selective races may not be relevant, but the information should be disseminated when issuing certificates of fitness, sports licenses, or integration courses for new runners.

### Electronic supplementary material

Below is the link to the electronic supplementary material.

Supplementary Material 1

## Data Availability

The data that support the findings of this study are available on reasonable request from INSERM laboratory U1042. Restrictions apply to the availability of these data, which were used under license for this study. Data are available from the authors with the permission of INSERM laboratory U1042. Please contact the corresponding author to request the data from this study.
