# Supplementary material for: Athletes’ medical preventive behaviors: the case of oral health and ultraendurance trail runners
Source: BMC Oral Health. 2024 Jul 11;24:777. doi: 10.1186/s12903-024-04492-3 (PMC11242021; doi:10.1186/s12903-024-04492-3)
Supplement: Supplementary file 1 — Supplementary Material 1 [file 12903_2024_4492_MOESM1_ESM.docx]

QUESTIONNAIRE (EN)

1/ Personal characteristics section

- Your gender:

M / F (single answer)

- Your age:

XX (open field)

- What is your nationality:

French / Spanish / Swiss / Italian / Other (single answer and free field for « other »)

- What is your socio-professional category (single answer)

Farmer/ Craftsman, merchant and company manager/ Managers and higher intellectual professions/ Intermediate Professions/ Employees/ Salaryman/ Retiree/ No activity for other reasons (single answer)

- How much time is dedicated to your work?

≤35h / week between 36-45h per week / > 45h per week (single answer)

- How is your general health? (only single answer possible)

Very good / Good / Fairly good / Poor / Very poor / Unkown (single answer)

2 / Sport habits section

- What is your ITRA Performance Index ?

XXX (open fields)

- How long have you been a trail runner?

<2 years / Between 2 and 5 years / Between 5 and 10 years / More than 10 years

(single answer)

- How much is your training volume over the last month?

≤ 3h per week / 3 to 7h per week / 7 to 12h per week / >12h per week (single answer)

- In what race do you participate on UTMB 2020?

UTMB / PTL / CCC / OCC / TDS / MCC (single answer)

3/ Eating habits section

- Do you take food besides meals and training?

Yes / No (single answer)

- Do you have solid or liquid food intake (excluding water) :

-During your short training sessions (less than 15 km or 60 min)?

Yes / No (single answer)

-During your long training sessions (more than 15 km or 60 min)?

Yes / No (single answer)

4/ Oral health section

- How is your dental health?

Very good / Good / Fairly good / Poor / Very poor / Unkown (single answer)

- How many missing teeth are not replaced?

Less than 4 missing teeth / 4 or more missing teeth (single answer)

- When was the last time you went to the dentist?

More than 1 year / Less than 1 year (single answer)

- Outside of this visit, you usually go to the dentist:

Only in emergencies / For a check-up (single answer)

- How often do you brush your teeth?

Less than twice a day / twice a day / more than twice a day (single answer)

- Have you implemented a dental hygiene technique while racing (gum, mouthwash, tooth brushing, etc…)?

Yes / No (single answer)
